# Increased sensitivity of sputum microscopy with sodium hypochlorite concentration technique: A practical experience at RNTCP center

**DOI:** 10.4103/0970-2113.76295

**Published:** 2011

**Authors:** Navinchandra M. Kaore, Kalpana P. Date, Vilas R. Thombare

**Affiliations:** *Department of Microbiology, Peoples College of Medical Sciences and Research Centre, Bhopal, India*; 1 *Department of Microbiology, NKP Salve Institute of Medical Sciences and Research Centre, Nagpur, India*

**Keywords:** Concentration, RNTCP, smear positivity, sodium hypochlorite, tuberculosis

## Abstract

**Background:**

In revised national tuberculosis control program (RNTCP), microscopic examination of sputum for AFB plays an important role in the initial diagnosis of tuberculosis. Bacillary concentration after decontamination and liquefaction by 5% sodium hypochlorite is useful in providing increased sensitivity and safety for handling of specimen.

**Materials and Methods:**

In this cross-sectional, prospective study, carried out at NKP Salve Institute of Medical Sciences and RC, Nagpur, patients were included according to RNTCP criteria. One set of smears was made according to the RNTCP guidelines while another set was prepared by concentration after decontamination with 5% sodium hypochlorite. Both set of smears were stained according to RNTCP method and were screened by two observers separately to remove observer’s bias and graded according to the RNTCP guidelines. A total of 591 sputum samples from 219 patients were included in the study with 168 males (76.71%) and 51 females (23.28%).

**Results:**

A total of 77 samples (13.02%) from 34 patients were positive by routine method whereas by concentration method 119 samples (20.13%) from 49 patients were found positive diagnosing 15 additional patients. This rise of 7.11% in sputum positivity over routine is highly significant (*P*=0.001021, χ^2^=10.78) with 44.11% increase in diagnosed cases.

**Conclusion:**

There is a statistically significant rise in smear positive cases after concentration with 5% sodium hypochlorite solution. Considering its low cost, decontaminating and liquefaction properties with better sensitivity, this method is safe and can be of vital importance; at least for smear negative cases.

## INTRODUCTION

India accounts for nearly one third of the global burden of tuberculosis. The major objectives of the tuberculosis control program are early detection and treatment of the infectious case of pulmonary tuberculosis.[1]

In revised national tuberculosis control program (RNTCP), microscopic examination of sputum for AFB plays an important role in the initial diagnosis of tuberculosis. The microscopic examination requires 10^4^ bacilli per milliliter of sputum in order to be detected on smear. Considering the amount of sputum material that is examined in oil immersion field, chances of missing the organism are high thus reducing the sensitivity. Much of the transmission of TB can occur even before the concentration in sputum reaches a critical level when it is diagnosed. A negative smear does not exclude the diagnosis of tuberculosis, as about 55% of pulmonary tuberculosis cases worldwide harbors low bacillary load. It has also been established that sputum smear microscopy is less sensitive in HIV –TB co infection where sputum smear tends to be negative.[2,3] Increasing the number of samples to be tested increases the smear positivity rate marginally when compared to three samples being tested in RNTCP.[4,5]

Pre-treatment of sputum by 5% sodium hypochlorite solution (NaOCl) ensures liquefaction of the sputum as well as disinfects it within 30 min without destroying the acid fastness of the mycobacterium tuberculosis.[6] Concentration of these pre-treated samples by centrifugation has been tried as a possible mean to increase the sensitivity of sputum direct microscopy by providing clean fields with less debris and making it safe for handling by laboratory workers.

With this background, we planned our study to look for increase in sensitivity of direct microscopy by concentration after pre-treatment with sodium hypochlorite over routine RNTCP method.

## MATERIALS AND METHODS

This cross-sectional study was carried out in the Department of Microbiology from February 2008 to June 2008. Patients of all ages and either sex coming to RNTCP center were included according to RNTCP criteria, having –

- Cough for three weeks or more duration.
- Known contacts of sputum smear positives irrespective of duration of cough.
- Extra pulmonary TB irrespective of duration of cough.

Attempt was made to collect three sputum samples (two spot and one morning sample) in a clean, wide mouth labelled container. Two sets of sputum smears were prepared from each sample.

For the first set, the mucopurulent portion of the sputum was taken on a new, clean and grease free glass slide and the smears were heat fixed and stained by modified ZN staining using 25% H_2_SO_4_ as a decolorizer.

In view of potential aerosol formation during manipulation of sputum samples for second set of smears, Bio-Safety Cabinet was used. 1-2 ml of sputum was taken in screw capped disposable test tubes. Equal volume of 5% sodium hypochlorite was added to the test tube and kept at room temperature for half an hour. The test tube was shaken intermittently after which approximately 8 ml of distilled water was added to the test tube and it was centrifuged at 3000 g for 15 min. The supernatants were carefully discarded and smears were prepared from the sediments.[7] The smears were heat fixed and stained similarly.

To remove observer’s bias, slides prepared by both the methods were observed by two experienced microbiologists separately by bright field microscopy and graded according to the RNTCP guidelines.

All the data was managed in Microsoft Excel and statistical analysis was done using Epi-info. Pearson’s chi-square test was used for comparative evaluation between two groups.

## RESULTS

**Figure 1:**
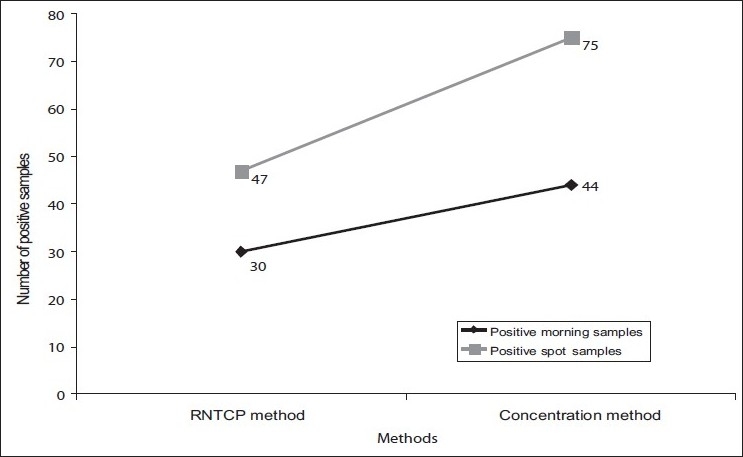
Positivity of morning and spot samples with different methods employed for smear preparation

A total of 219 patients were included in the study with 168 males (76.71%) and 51 females (23.28%), a ratio of 3.29 to 1. Totally, 591 sputum samples collected from the patients were studied. From all the 219 patients at least two samples were collected, one spot and one morning sample. Of the total 591 samples, 77 (13.02%) samples (30 morning and 47 spot) were positive by routine method employed by RNTCP, whereas by concentration method 119 samples (44 morning and 75 spot) (20.13%) were found positive, thus an increase of 14 in morning samples and 28 in spot samples from RNTCP method. When compared with the RNTCP method the increase of 7.11% over routine is highly significant with *P*<0.001 (χ^2^ =10.78) [Table 1 and Figure 1].

**Table 1:** Comparative evaluation of sodium hypochlorite concentration method over RNTCP method

	Samples positive	Samples negative	Total	
Concentration method	119	472	591	Chi-square value = 10.78
RNTCP method	77	514	591	*P* = 0.001021
Total	196	986	1182	

A total of 34 patients were diagnosed by RNTCP method. Additional 15 patients were diagnosed using concentration method which otherwise would not have been diagnosed by RNTCP method, as shown in Table 2. Out of these 15, 3 patients which were negative by routine method, and one smear positive by concentration method showing grading of 1+ were subjected to X-ray examination as per guidelines of RNTCP and were reported as radiologically positive for tuberculosis.

**Table 2:** Comparison of smear positivity in additional 15 patients diagnosed by sodium hypochlorite method against the routine RNTCP method

Additional patients diagnosed over RNTCP (*n*=15)	Status by RNTCP method	Status by sodium hypochlorite method	Grading in sodium hypochlorite method	Remarks
3	Smear negative	One smear positive	1+	Patients had positive X-ray findings (treatment initiated)
5	Smear negative	Two smears positive	2+	Treatment initiated
7	One smear positive	Two smears positive	4/7 – 2+ 3/7 – 3+	Treatment initiated (X-ray not required)

Five smear negative patients by RNTCP method were smear positive in two smears by concentration technique with a grading of 2+ and were put on DOTS.

Seven patients were one smear positive by routine RNTCP method but were two smears positive by concentration technique with a grading of 2+ in four out of seven patients and 3+ in rest, thus obviating the need for X-ray examination.

These 15 additional cases diagnosed constituted to 44.11% increase in case detection when compared to RNTCP method. Thus concentration method is highly sensitive.

Of the 77 samples positive by routine method, 47 were spot samples and 30 were morning samples. By concentration method, there is significant increase in spot samples diagnosed as positive from 47 to 75 and morning samples from 30 to 44 [Figure 1].

Both the observers reported clear fields with less debris in smears prepared after sodium hypochlorite treatment as well as significant increase in average number of AFB seen per field thus making it less strenuous for the observers.

## DISCUSSION

Direct microscopy of sputum is currently the backbone for diagnosing pulmonary tuberculosis in national programs particularly in low-income countries. It is a rapid, inexpensive and highly specific method for the detection of AFB in sputum. Major disadvantage is the discouragingly low sensitivity when used in overburdened control programs like the RNTCP.

The study aimed at increasing the sensitivity of paucibacillary samples by concentration after pre-treatment with sodium hypochlorite which also makes sputum samples safe to be handled by laboratory workers in RNTCP setup.

Being a tertiary care hospital having the BSC (Bio Safety Cabinets), we have used the screw capped bottles and transferred the sputum sample from the original wide mouth container in which it was collected and added the sodium hypochlorite to it. Alternatively, sodium hypochlorite can be added to the original wide mouth container itself which will liquefy the sputum and will also disinfect the sample within 30 min,[6] so that it will be safe to be handled by laboratory workers even at places where BSC is not available.

RNTCP manual states that average 10% of TB suspects are expected to have sputum smear positive.[3] In our study, 13.02% of suspects were sputum smear positive by routine method whereas 20.13% were positive by the concentration method after treatment with sodium hypochlorite. Similar findings had been reported by Hakan Miorner of Ethiopia 1996[7] and Saxena *et al*. 2001.[1]

In our study, we observed an overall increase of sputum smear positivity by 7.11% by concentration method. Makunde WH *et al*. 2007 from Tanzania has reported an increase of 15.6% in smear positivity by this concentration method as compared to routine method.[8]

In our study, majority of the smear positive samples in 219 patients enrolled were either spot and morning samples, obviating the need for the third sample being tested as being done in RNTCP now as it yield very poor results.

In our study, there were 15 additional cases diagnosed over 34 cases diagnosed by routine RNTCP method which is marked increase of 44.11% over the routine. Three patients having one smear positive required X- ray examination to confirm the diagnosis while in 12 patients X-ray was not needed as they had two smears positive by concentration method while by RNTCP method the smears were either negative or only one was positive. All these 15 patients got the benefit of early detection and prompt treatment which other wise would have gone undetected as open cases spreading infection in the community.

Of the total samples processed, there was substantial increase in smear positivity in spot samples from 47 to 75 when samples were concentrated after sodium hypochlorite treatment, which is attributed to concentration as well as clearer fields with less debris. The smear positivity in morning samples has always been more as compared to spot samples and that might be the reason for marginal increase in the smear positivity of morning samples after concentration from 30 to 44.

Use of sodium hypochlorite for pre-treatment before concentration is not labor intensive and could be carried out with same technical team with additional requirement of centrifuge machine only.

If not all, only those samples which are negative by routine RNTCP method can be retested by sodium hypochlorite concentration method. This can be done on the same day as procedure requires only half hour of pre-treatment with time required for centrifugation and staining. Thus results can be given with a delay of not more than 24 h for all sputum smear negative patients. A substantial increase of 44.11% in the new case detection in our study definitely shows that improved sensitivity compensates for a 24 h delay in the reporting.

To conclude, increased sensitivity of microscopy after treatment with 5% sodium hypochlorite solution is due to concentration, increasing the number of bacilli per fields as well as clean fields with less debris. Sodium hypochlorite is cheap, easily available and also included in the list of chemicals used in RNTCP. Also, sodium hypochlorite liquefies the purulent sputum samples and increases detection rate. The decontaminating property of sodium hypochlorite has an advantage of limiting laboratory infections. This method of concentration of sputum after treatment with sodium hypochlorite can be of vital importance; at least for samples which are coming negative with routine methods. Majority of the designated microscopic centers (DMC) under RNTCP do not have centrifuge or Bio-safety cabinets but in absence of new and better tool which could replace sputum microscopy in the near future; increased sensitivity of sputum microscopy by this method may need to be validated for feasibility and applicability under field conditions by large-scale studies and can certainly be implemented in institutions with facilities for centrifuge.
